# A link between age, affect, and predictions?

**DOI:** 10.1007/s10433-022-00710-5

**Published:** 2022-07-05

**Authors:** Sabrina Trapp, Marc Guitart-Masip, Erich Schröger

**Affiliations:** 1 Macromedia University of Applied Sciences, Munich, Germany; 2 Aging Research Center, Karolinska Institutet, 17165 Stockholm, Sweden; 3 Centre for Psychiatry Research, Department of Clinical Neuroscience, Karolinska Institutet & Stockholm Health Care Services, Stockholm, Sweden; 4 Max Planck University College London Centre for Computational Psychiatry and Ageing Research, University College London, London, WC1B 5EH UK; 5 Wilhelm Wundt Institute for Psychology, Leipzig University, Leipzig, Germany

**Keywords:** Predictive coding, Affect, Top-down, Bayesian brain, Psychiatry, Prior

## Abstract

The prevalence of depressive symptoms decreases from late adolescence to middle age adulthood. Furthermore, despite significant losses in motor and cognitive functioning, overall emotional well-being tends to increase with age, and a bias to positive information has been observed multiple times. Several causes have been discussed for this age-related development, such as improvement in emotion regulation, less regret, and higher socioeconomic status. Here, we explore a further explanation. Our minds host mental models that generate predictions about forthcoming events to successfully interact with our physical and social environment. To keep these models faithful, the difference between the predicted and the actual event, that is, the *prediction error,* is computed. We argue that prediction errors are attenuated in the middle age and older mind, which, in turn, may translate to less negative affect, lower susceptibility to affective disorders, and possibly, to a bias to positive information. Our proposal is primarily linked to perceptual inferences, but may hold as well for higher-level, cognitive, and emotional forms of error processing.

## The brain as a prediction device

One dominant idea in cognitive neuroscience is that the brain is proactive, i.e., it processes information based on expectations or predictions. Several recently developed, large-scale frameworks such as *Predictive Coding*, *Bayesian brain*, and *Free-energy principle* share the key idea of the brain as a “prediction device” (for a review see Clark 2013). In all these frameworks, it is assumed that the physical and social environment and its structures are represented by the brain as a *generative model*. This internal model allows making predictions about likely interpretations of incoming data, and studies demonstrated that such predictive information is already activated before actual stimulus input (Blom et al. 2020, Trapp et al., 2015). Deviations of such predictions, the *prediction errors*, help to iteratively refine these models and update according to changes in the world. The rise of the predictive brain frameworks stimulated numerous studies designed to detect correlates, networks, and consequences of predictions and prediction errors (e.g., Alink et al., 2010; de Gardelle et al. 2013; Todorovic et al. 2011). Furthermore, researchers started to re-interpret symptoms of clinical disorders as possible deficiencies in predictive processing. For example, schizophrenia has been re-explained by deviant prediction error signaling (Horga et al. 2014; Umbricht & Krljes 2005). Anxiety is an emotion that is inherently linked to the future, i.e., by expecting aversive things to happen, and has therefore consequently been associated with biased prediction processes (Grupe & Nitschke 2013; Trapp & Kotz 2016). Autism has been linked to aberrant predictive processes, in that these individuals perceive the world based on fewer expectations, thus are “flooded” with raw data from bottom-up, or handle prediction errors less flexibly (Pellicano & Burr 2012; van de Cruys et al., 2014).

What can such frameworks contribute to understanding the aging brain, and particularly, to the altered affective states observed in this population? Older adults tend to show more positive and less negative affect, an overall increase in subjective well-being, and a bias to positive stimuli (Cacioppo et al. 2008; Charles et al. 2001; Mather & Carstensen, 2005; Stawski et al. 2008). Multiple psychological explanations have been proposed, such as more emphasis on and greater resource allocation to emotion regulation (Carstensen 1993; Knight et al. 2007), better control over emotions (Gross et al. 1997; Phillips et al. 2008), or less regret (Brassen et al. 2012). Furthermore, the prevalence of depressive symptoms decreases from early to middle age adulthood (Fiske et al. 2009). Here, we suggest that older individuals show more subjective well-being, are biased to positive information, and healthy, middle-aged individuals are less susceptible to depression, because they generate attenuated prediction errors.

## Age and predictions

Although we do not know how the world is subjectively experienced by a newborn baby, one can assume that it is rather chaotic, because there are considerably less expectations of the world yet when compared with children and adults. Over time, we acquire and exploit regularities of our world: For instance, leaves will usually reside on the top of a tree; a giraffe rarely would appear in a kitchen, and the sequence of the colors of the traffic light is predictable as well. Such temporal and spatial regularities and associations help the brain to generate predictions about what is coming next (Bar 2007). What would one expect to change with age? Intuitively, longer exposure to environmental regularities of our physical and social world allows incorporating more prior information, harbored in the brain as a generative model, stored in long-term memory. Thus, reliance upon top-down information could increase with age. Support for such a more prominent usage of top-down information comes from several studies. In an object recognition task that contained perceptual ambiguity, behavioral data demonstrated that older adults benefited more from contextual information, and showed higher reliance on top-down processes—this was reflected on the electrophysiological level as well (Lai et al. 2020). There also is evidence for stronger reliance on top-down information from functional magnetic resonance imaging (fMRI): Gilbert and Moran (2016) fitted three different brain connectivity models to data from an object-naming task—the included brain areas were the early visual cortex, anterior ventral temporal cortex, angular gyrus, and inferior frontal gyrus. Data from healthy older individuals showed earlier and higher evoked frontal activity, and a better fit with models that incorporated predictive coding principles. Moreover, there is evidence that model updating is attenuated in aged brains, suggesting less sensitivity to bottom-up signals (Moran et al. 2014). One important brain index of predictive processing is the Mismatch Negativity (MMN), an event-related potential assessed with electroencephalography (EEG). It usually occurs as the brain´s response to sensory events that *deviate* from a prediction, e.g., by violating a rule inherent in a series of stimuli (Wacongne et al. 2012; Winkler & Schröger 1995). The MMN is regarded as either directly reflecting prediction error processing or model-updating, a process that re-calibrates current models of the environment based on incoming sensory data. Thus, it can be conceived as being at least an indirect indicator of prediction error processing. A meta-analysis revealed that many studies with aged individuals reported an *attenuation* of the MMN amplitude, and some showed an increase in the MMN latency (Cheng et al. 2013). These data support the idea of modulated prediction error processing in the aged mind.

The bottom-up to top-down shift of brain activity in aged individuals has previously been interpreted as a compensatory mechanism to maintain cognitive performance. In this framework, the prefrontal cortex is suggested to take over to compensate for the functional decline of posterior brain regions which are associated with the processing of sensory information (Davis et al., 2008). However, this interpretation has been challenged by a Bayesian account of life span development. This alternative interpretation posits that as the brain ages (from early adulthood on), it holds a better model, and is increasingly dominated by top-down predictions, which in turn allow for decreased complexity of the models and their implementation in the brain (Moran et al. 2014). Decreased complexity may translate into a brain that naturally decreases the density of neuromodulators, gray matter volume and white matter connections, because the models of the environment it holds gets more certain. The idea that better models of the environment allow for decreased complexity of their biological, neuronal implementation is indirectly supported by experimental evidence: highly trained ballet dancers exhibit *reduced* gray matter volume in cerebellar regions as a result of *increased* motor skills (Nigmatullina et al. 2015). Similarly, when training motor skills, gray matter volume in the motor cortex increases early on during training, but re-normalizes after some weeks, despite continued motor improvement (Wenger, 2017). Such data suggest that the age-related brain shrinkage typically observed may be to some extend adaptive and an indicator for growing expertise or a better model of the world. Of course, it is possible that there are two parallel processes involved in the age-related shrinkage of brain structures: One that is adaptive and that potentially starts early on at the end of adolescence, i.e., as the predictive models stabilize and the brain undergoes synaptic pruning (Crews et al. 2007; Selemon 2013), and one that is pathological and linked to age-related vascular and inflammatory changes (Raz and Rodigue 2006; Pini et al 2016). The pathological process may be linked to a process of disintegration and a deteriorating model of the world, which may for example underlie dementia and late onset depression.

## Predictions and affect

From a biological point of view, organisms are driven by survival relevant approach or avoidance behavior (Panksepp 2008). If accurate predictions are one of the brain’s major computational goals (Friston 2010), would it not be useful if successful surprise minimization (with the reduction of prediction error as a proxy) is itself rewarding or hedonically valuable, and conversely, a failure herein aversive? Ramachandran and Hirstein (1999) already suggested that perceptual inferences must be linked to limbic brain systems to provide incentive for discovering image correlations, binding, grouping, and identifying content. A variety of recent theoretical proposals and studies advocate an *affective tagging* of perceptual predictive processing. For instance, Chetverikov and Kristjansson (2016) proposed that a fulfilled perceptual prediction is associated with positive affect, while deviations or prediction errors elicit negative affect. The authors re-interpret several human choice data, such as preference for symmetry, sharpness, contrast, and coherency as a function of stimulus predictability. Van de Crys (2017) linked  valence and the rate of prediction error reduction. Positive affect, in this account, is equivalent to a positive prediction error reduction rate, and conversely, negative affect to a less effective prediction error reduction rate.

There is evidence for affective consequences of successful perceptual predictions from several studies. Associations in the natural world help to generate predictions, e.g., the context of a bathroom is associated with objects such as towel, shower, and toothbrush (Bar 2007). Trapp et al. (2015) used artificial visual stimuli that were regularly associated with others vs. stimuli that were only randomly assigned to other stimuli. In a subsequent surprise forced-choice preference task, participants significantly preferred the associative stimuli over the random stimuli, although both stimuli were equally familiar to participants. A study by Ogawa and Watanabe (2011) used a contextual cueing paradigm, and participants had to discover a target among distractors. The distractors were either predictive of the target or were randomly distributed and could thereby not predict the location of the target. When eventually asked which layout they prefer, participants significantly chose the predictive over the random layout. Braem and Trapp (2019) showed that when exposed to a series of subsequently presented stimuli with hidden pairs, i.e., one stimulus predicted the occurrence of another, participants preferred the stimulus that had helped them to make predictions over random and over the predicted stimulus. This could indicate that facilitated predictive processing is tagged as inherently positive. Ryali et al. (2020) asked participants to rate several pictures of faces for how attractive and positive they appeared. The authors found that the judgments linearly increased with the statistical typicality of the faces. There are various explanations for this effect, but the authors suggest one reason could be that statistical atypicality is related to “unexpected uncertainty.” Bianco et al. (2019) manipulated the predictability of melodies and found that pupil dilation was particularly linked to predictable melodies, indicating greater arousal and hedonic pleasure.

Ultimately, continuous experience of faulty expectations and consequently, a large amount of prediction errors may even result in a manifest clinical affective disorder. It has already been suggested that depression is accompanied by erroneous updating of predictions, which in turn increase and sustain feelings of uncertainty and loss of control (Schutter 2016). The preservation of depressive symptoms, in turn, has been explained by active inference, i.e., active sampling of the world according to one’s expectations, which in case of depressed patients only fulfill the negative prophecies (Chekroud 2015). The MMN may also unravel potential links between depressive symptoms and prediction mechanisms. Some studies report an increase in MMN amplitude in participants suffering from depression (e.g., Kahkonen et al. 2007) and participants being at risk for depression (Boneti et al. 2017), indicating higher prediction error processing in this affective disorder. However, others found a decrease in the MMN amplitude (e.g., Hirakawa et al. 2017; Naismith et al. 2012). Although the effect of depression on prediction error processing as indicated by MMN can go in two opposite directions, the MMN-depression studies convincingly show that sensory predictive processing is altered in depression. As the experimental paradigms used in the MMN-depression studies utilized different kind of rules and rule violations (e.g., pitch or intensity), different oddball paradigms (e.g., controlling for refractory effects or not), different kind of populations (e.g., major depression, first-episode, at-risk, type of comorbidity) and different types of how to control for drug effects, the critical factor(s) determining increase or decrease in MMN amplitude are yet unknown.

Although studies using perception paradigms suggest a clear link between successful prediction and positive affect, there is evidence that prediction errors may also have a positive influence on affect in the context of reinforcement learning paradigms. Specifically, it has been shown that happiness while performing a gambling task can be predicted from the magnitude and sign of the reward prediction errors induced by the task: happiness ratings increased and decreased following positive and negative prediction errors, respectively (Rutledge et al. 2014). Interestingly, the amplitude of the BOLD response in the ventral striatum, a region presumably reflecting dopaminergic afferents conveying prediction errors (O'Doherty 2003), also predicted happiness, and the effects of prediction errors on happiness were amplified after a pharmacological intervention that boosted dopamine levels (Rutledge et al. 2015). It is important to note, however, that there is a fundamental difference between reinforcement learning and perceptual tasks. In the context of reinforcement learning,the reward prediction error, i.e., the difference between expected and obtained reward, is linked to an externally given reward. In this sense, positive reward prediction errors are good news because an unexpected reward is given, and negative reward prediction errors are bad news indicating that the world is worse than expected. These *signed* error signals are used to update the expectation and support the goal to maximize reward and minimize punishment through action selection in an uncertain environment (Sutton & Barto 1998). On the other hand, prediction errors emerging in low-level information processing in perception and action lack such externally given valence. Instead, as reviewed above, in perception, it is the correctness of the prediction itself that may be interpreted as a reward by the brain. Furthermore, there is no “positive prediction error” in perception and action: A prediction about an upcoming movement or sensory input is either correct or incorrect. Accordingly, while several commonalities exist, reward prediction errors differ from sensory prediction errors in many aspects, and thus may be differently associated with affect. Although we focus on sensory prediction errors in this proposal, we do not imply that the mechanism we propose here is the only valid computational account of how fluctuations in affect or depression may emerge. In fact, multiple suggested reinforcement learning mechanisms have been described to explain mood fluctuations (Eldar and Niv 2015; (Rutledge et al. 2014) and even depression (Bishop and Gagne 2018; Huys et al. 2015). We suggest that several of these mechanisms may operate in parallel.

## A link between age, affect, and predictions?

The prevalence of subthreshold depressive symptoms decreases from younger to middle age (Sutin et al. 2013). Furthermore, there is considerable evidence that emotional well-being increases with age. In two studies by Carstensen and colleagues (Carstensen et al. 2000, 2011) participants had to report their emotional states at five times each day for a one-week period. This procedure was repeated five and ten years later. The results showed that negative emotions were less frequent, and periods of positive emotional experiences lasted longer than negative emotions among older adults. Furthermore, they reported that age is associated with an improvement of overall emotional well-being, with greater emotional stability, and with more emotional complexity (i.e., co-occurrence of positive and negative emotions). The authors interpreted their findings by the *Socioemotional Selectivity Theory* (Carstensen et al. 2003), and suggest that people change their motivational goals and strategies throughout life span. They argue that older people have an increased motivation to maintain emotional balance and affective well-being. In addition, older individuals seem to be biased toward positive information, termed the *positivity effect* or bias (Carstensen, & Mikels 2005; Mather, & Carstensen 2005). In a study using a dot-probe paradigm, for instance, a neutral and a negative face were presented on either side of the computer screen (Mather, & Carstensen 2003). After the faces disappeared, a dot was presented on the location of one former stimulus, and participants had to detect the dot as quickly as possible. Older participants responded faster if the dot appeared on the same side as a neutral face. Gaze patterns when viewing negative and positive faces demonstrated that older individuals’ attention was directed toward the happy face, and away from the angry face, whereas young adults did not show this attentional preference (Isaacowitz et al. 2006). These findings reflect an attentional bias away from negative and toward positive information in older individuals.

We propose that middle age and healthy older adults produce attenuated prediction errors and suggest that these attenuations result in more positive and less negative affect, and lower susceptibility to affective disorders. There is considerable literature on the link between positive affect and resilience to depression—for brevity, we will not review this here (e.g., Burgdorf et al. 2017; Cohn et al., 2009; Fredrickson et al. 2003; Lyubomirsky et al., 2005). If predictions in the aged mind are linked to attenuated prediction error processing, and if this results in a positive feedback, this may also explain the positivity bias. Important to note is that the exact causes for this bias are still subject to intense discussions. One explanation is rather motivational in nature, i.e., that aged individuals realize that their life horizon is constrained, and accordingly shift their priorities and resources to emotional satisfaction (Carstensen et al. 2003). Others propose that the bias to positive information in aged individuals is due to positive stimuli being less cognitively demanding, or by aged individuals having reduced neural and affective responses to negative stimuli due to impairments in the amygdala (Labouvie-Vief et al., 2007; Cacioppo et al. 2011). Possibly, a positive affect elicited by attenuated prediction error processing, may influence the content or valence of working memory (WM) content temporarily. The content of WM is considered as a top-down template that guides attention (Downing 2000; Fockert et al. 2001). Thus, the positive affect may guide and bias attention toward positive information in the external world.

We deliberately remain agnostic regarding the exact modulations of predictive processing in the aged mind, as conclusions or even hypotheses at this stage would clearly be premature. There are studies that report a greater reliance upon predictions with age, and studies that show the MNN, an index of prediction error processing, to be attenuated. Although there is a good reason to assume that the generative models of elderly are more accurate (due to longer experience with the world), and accordingly, result in *better* predictions, it could also be that the aging brain is not able to detect deviations appropriately, and the brain mistakes this as a successful prediction. The modifications can be motivational in nature or a result of, for instance, attention being more distracted with age, thus making these individuals less efficient in detecting and processing errors in the first place. In this context, it should also be examined whether the attenuated MMN effects in the elderly are due to modulated predictive processes or to slowed deviance-detection mechanisms (i.e., the computation of the prediction error), which would fit to research revealing impaired attentional orienting functions in elderly. However, important to note is that the *result* of these various possibilities remains the same: prediction error processing is attenuated. Certainly, the exact modulations of predictive processsing and the underlying affective states remain an important route for future scientific investigation.

The above-mentioned studies suggest that older age is both associated with biases of attention to positive information and modulated emotional regulation that sustain positive affect. These effects may be reasons for decreased risk of depression in older age. However, this view is only partially supported by epidemiologic studies on depression across the lifespan. Whereas the prevalence of depression tends to decrease after the age 65 (Haigh et al. 2018) and is threefold higher in 18–29 year old individual than in individuals aged 60 or older (DSM-V), there is discussion in the literature on whether the current diagnostic criteria for major depression are ill suited to capture the disorder in older adults (Haigh et al. 2018). In fact, the prevalence of subthreshold depressive symptoms decreases from younger to middle age (Sutin et al. 2013) but increases again in older adults (Fiske et al., 2003; Luppa et al. 2012; Sjoberg et al. 2017; Sutin et al. 2013). How can positive affect and subthreshold depressive symptoms simultaneously increase in older adults? One reasonable possibility is that only the older adults who do *not* experience any subthreshold depressive symptoms show increased positive affect, and that previous studies have focused on this subpopulation. Furthermore, it is possible that the increase in subthreshold depressive symptoms in older adults reflects a process distinct from the depression observed in younger adults. Major depression is a chronic recurring disorder that normally starts early in life, with a peak occurrence of a first episode around the end of adolescence and beginning of adulthood (Association, 2013). However, a substantial proportion of older patients with depression experience the disorder for the first time, known as late onset depression (Fountoulakis et al. 2003). It is discussed that these two peaks of onset of depression represent two distinct clinical entities. Compared to the depression that debuts in adolescence, late onset depression has less of a genetic component, is more associated to structural changes in the brain and entails higher risk for dementia later in life (Fiske et al. 2009). In consideration of this evidence, it is possible that older participants whose brain stays healthy show increased positive affect in their development. Those individuals would mainly show structural brain shrinkage associated with an altered generative model, as discussed above. In contrast, accumulated pathological changes in the brain may disrupt the anatomical substrate of the predictive models. This would eventually result in increased prediction errors, and consequently, in negative affect or even depression. Importantly, the hippocampus, one of the first regions which is affected in Alzheimer dementia,  with a decline in neurogenesis (Brueggen et al., 2015; Moreno-Jiménez et al. 2019), also seems to be an important tool for predictions: the region CA3 generates recurrent activity, which is transferred to the visual cortex. By using a pattern classification method, Hindy et al. (2016) found that the hippocampus contained the mnemonic information required for predictions in a sequence learning task. Thus, aberrant predictive processing may be another important symptom accompanying dementia. In line with this idea, when subsequent sounds were presented with some of them being deviant to the sequence, with long inter-stimulus intervals, only data from healthy elderly participants showed the MMN, but not data from elderly participants with Alzheimer dementia (Ruzzoli et al. 2016).

## Summary and outlook

We here suggested a link between altered predictive processing in elderly, affect and depression. Specifically, we propose that the bias to positive information in elderly and decreased depressive symptoms in middle-aged individuals may be linked to attenuated prediction error processing. Note that our proposal holds for all kind of sensory information, i.e., we also expect that predictions errors are attenuated in the auditory or gustatory domain. However, we by no means imply a direct and necessarily causal link between less negative affect or more positive affect and resilience to depression. There are various cognitive, emotional, genetic, and physiological factors that may act as modulators here.

While current data are in principle compatible with the idea that alterations in predictive processing are associated with a stronger resilience against depressive symptoms in middle age and older adults, this hypothesis is currently not warranted by direct empirical evidence. Such support would come from longitudinal studies investigating predictive functions and depression in different age groups. One would predict that the brain parameters reflecting top-down processes are negatively correlated with depressive symptoms. Conversely, a stronger reliance on top-down processes in younger, clinically depressed patients could be positively related to severity and resistance to treatment, as in this case, it may reflect more resistant to changes of their (faulty) model of the world.

If our proposal is valid, predictive processing may serve as a neurophysiological marker that can be used for early detection of depressive symptoms. Appropriate predictors of therapy outcome for depression are still lacking (Amick et al. 2015). Given our assumptions are correct, providing insight into the predictive capabilities of patients may fill this gap. Finally, our proposal could ultimately guide the development of age-related depression prevention programs, or help building better treatments for midlife depression. If prediction error processing is  linked to affective well-being in the aging mind, one may develop training programs that support the acquisition and usage of regularities, from low-level sensory processing, such as visual and auditory stimuli, to higher-level expectations that may built upon aquired semantic knowledge.
